# Rapid review of gender-affirming healthcare for children and adolescents: Evidence synthesis (2021–2025) and recommendations for South Africa

**DOI:** 10.4102/sajhivmed.v27i1.1800

**Published:** 2026-08-28

**Authors:** Ingrid Lynch, KL Dunkle, Chris McLachlan, Pierre Brouard, Landa Mabenge, Sakhile Msweli, Liberty Matthyse, Marion Stevens, Robin Dyers, Jenna-Lee de Beer-Procter, Kevin Adams, W.D. Francois Venter, Elma de Vries

**Affiliations:** 1 Department of Critical Studies in Sexualities and Reproduction, Rhodes University, Makhanda, South Africa; 2 Centre for Sexualities, AIDS and Gender, University of Pretoria, Pretoria, South Africa; 3 Hubert Department of Global Health, Emory University, Atlanta, United States; 4 KwaZulu-Natal Department of Health, Pietermaritzburg, South Africa; 5 Department of Psychology, University of South Africa, Pretoria, South Africa; 6 Division of Public Health Medicine, School of Public Health, University of Cape Town, Cape Town, South Africa; 7 KwaZulu-Natal Department of Health, Empangeni, South Africa; 8 Gender DynamiX, Cape Town, South Africa; 9 Department of Political Science, Stellenbosch University, Stellenbosch, South Africa; 10 Division of Health Systems and Public Health, Department of Global Health, Stellenbosch University, Cape Town, South Africa; 11 WHO-FIC Collaborating Centre, Burden of Disease Research Unit, South African Medical Research Council, Cape Town, South Africa; 12 Department of Health and Wellness, Western Cape Government, Cape Town, South Africa; 13 Department of Psychology, Stellenbosch University, Stellenbosch, South Africa; 14 Division of Plastic, Reconstructive and Maxillo-Facial Surgery, Faculty of Health Sciences, University of Cape Town, Cape Town, South Africa; 15 Wits Ezintsha, Faculty of Health Sciences, University of the Witwatersrand, Johannesburg, South Africa; 16 School of Medicine, Faculty of Health Sciences, Nelson Mandela University, Gqeberha, South Africa

**Keywords:** gender-affirming healthcare, transgender and gender diverse, transgender health, gender affirmation, psychosocial support, gender-affirming hormone therapy, gender-affirming surgery, South Africa, sexual health, HIV prevention

## Abstract

**Background:**

Since the publication of the Southern African HIV Clinicians Society Gender-Affirming Healthcare (GAHC) Guideline in 2021, global evidence on care for transgender and gender-diverse (TGD) youth has expanded. An updated, locally grounded, evidence-informed assessment of health outcomes can support South African stakeholders, particularly as both adolescents and TGD persons are populations of relevance to HIV prevention, sexual health, and integrated healthcare programmes in South Africa.

**Objectives:**

To synthesise global empirical evidence (2021–2025) across psychosocial, endocrine, surgical, policy, and non-medical gender-affirming interventions for TGD youth under 18, with attention to South Africa’s social, legal, and health-system, and HIV-service delivery context.

**Method:**

A rapid review was conducted across 12 databases, supplemented by targeted searches for recent systematic reviews. Eligible sources comprised peer-reviewed empirical studies (*N* ≥ 5) reporting psychosocial or physical health outcomes of relevant interventions for TGD youth under 18 (or their families), and systematic or grey-literature reviews with reproducible methods. Findings were synthesised narratively by intervention domain.

**Results:**

The review included 200 primary studies, 29 academic systematic reviews, and four grey-literature reviews. Affirming psychosocial interventions were associated with reduced distress, anxiety and suicidality, and improved functioning and belonging. Puberty blockers and hormone therapy produced expected physiological outcomes under specialist care, with generally mild adverse events and stable or improved mental health. Restrictive policies were linked to poorer mental health, while protective policies improved outcomes. Most studies were small, observational, and Global North-focused.

**Conclusion:**

Evidence supports the safety and effectiveness of GAHC for TGD youth. Strengthening affirming care, timely clinical access, and protective policies in South Africa may improve healthcare engagement and support the delivery of integrated adolescent, sexual health, and HIV-related services for TGD youth.

**What this study adds:** This rapid review of evidence indexed January 2021 to August 2025 indicates that GAHC for TGD youth improves well-being, reduces psychological distress, prevents harm, and supports healthier developmental and mental-health outcomes. Findings inform South African HIV and sexual health practice and policy. This review updates the evidence underpinning the GAHC Guideline and provides clinically relevant evidence for multidisciplinary teams delivering HIV, sexual, reproductive, and adolescent healthcare services.

## Introduction

Transgender and gender-diverse (TGD) is an umbrella term referring to people whose current gender identities do not align with the sex assigned to them at birth.^1^ Adolescents within this group experience some of the greatest barriers to accessing healthcare globally.^2^ In South Africa, research has documented a high prevalence of HIV among transgender women,^3^ substantial healthcare access barriers for TGD people,^4,5^ and evidence that access to gender-affirming hormone therapy (GAHT) is associated with improved engagement across the HIV continuum of care, including viral suppression.^6^ The National Strategic Plan for HIV, TB and STIs 2023–2028 identifies both adolescents and TGD persons as priority populations for interventions, and calls for the inclusion of gender-affirming services across all levels of care.^7^

Since publication of the 2021 Southern African HIV Clinicians Society (SAHCS) Gender-affirming Healthcare (GAHC) Guideline,^8^ the evidence base on GAHC for children and adolescents has expanded substantially. This body of literature spans a wide range of study designs, scopes, and quality, and has been generated predominantly in Global North settings. In parallel, numerous systematic reviews,^9,10,11,12,13,14,15,16,17,18,19,20,21,22,23,24,25,26,27,28,29,30,31,32,33,34,35,36^ evidence syntheses,^37,38,39,40^ and updated international clinical guidelines^1,41,42,43,44,45^ have been released. Taken together, these developments underscore a pressing need among South African stakeholders – including TGD youth, their families and caregivers, educators, mentors, and clinicians – for a comprehensive, rigorous, and transparent synthesis of emerging evidence that is interpreted through local constitutional, historical, and socio-cultural commitments. These commitments shape health policy, clinical practice, and rights-based care in South Africa, including constitutional protections for gender identity as affirmed through equality jurisprudence.^46,47,48^ While global debates are often shaped by political and cultural dynamics specific to the Global North,^49,50,51,52,53^ South Africa must assess emerging evidence in relation to its own constitutional and public health frameworks, including the principles of Ubuntu, Batho Pele, equity, and collective well-being.^54,55,56^ Moreover, the South African context is shaped by health system inequities – including medication stock-outs, provider shortages, and the concentration of services in urban tertiary centres – that affect access to and continuity of care.^4,57,58,59^ These constraints are critical to interpreting the global evidence base within South Africa.

The conceptualisation of this review arose through collaboration between the SAHCS, as custodians of the 2021 GAHC Guideline^8^; original authors of the Guideline; Gender DynamiX, a South African transgender advocacy organisation that partnered in developing the Guideline; and the Professional Association for Transgender Health South Africa (PATHSA), which was formed in 2020. PATHSA endorsed the Guideline in 2021 as a professional body supporting the development and stewardship of GAHC practice in South Africa.

This independent rapid review synthesises research indexed from 01 January 2021 to 14 August 2025 to evaluate whether new evidence supports updates to or refinements of the 2021 SAHCS GAHC Guideline.^8^ Its overall aim is to collate findings from recent empirical research to support evidence-informed South African clinical practice and the development of a future guideline intended to supersede the 2021 SAHCS GAHC Guideline. The review was conducted by a gender and sexually diverse South Africa-based team with methodological, clinical, and lived-experience expertise. This perspective informed interpretation of retrieved evidence through an equity-oriented, rights-based, and context-sensitive lens. Full partnership with knowledge users strengthens evidence synthesis^60^ by ensuring that analyses engage with realities of unequal access, historical trauma, and structural barriers that characterise healthcare experiences for many TGD young people in South Africa.^4,5,6,61,62^

### Objectives

The review sought to:

- Synthesise global empirical evidence indexed between January 2021 and August 2025 across all types of GAHC interventions for TGD youth under 18, with attention to both benefits and potential harms.
- Assess the alignment between emerging evidence and the 2021 SAHCS GAHC Guideline, identifying where new findings support, refine, challenge, or extend existing recommendations.
- Interpret global findings within South Africa’s constitutional, health-system, and socio-cultural context, including historical inequities and intersecting structural barriers – such as poverty, violence, stigma, racism, sexism, xenophobia, homophobia, transphobia and cisnormativity – that shape health risks, access to care, and lived experience.

In doing so, this rapid review aims to support healthcare providers, policymakers, educators, civil society, and families in delivering care that is evidence-informed, developmentally appropriate, and aligned with the rights and dignity of TGD youth.

## Research methods and design

A rapid review approach was adopted to balance methodological rigour with timeliness.^60,63,64^ Rapid reviews apply systematic review principles while streamlining elements such as screening processes and data extraction to produce timely, policy-relevant evidence within constrained timeframes.^63^ This approach was selected to inform planned updates to the 2021 GAHC Guideline while maintaining transparency and analytic integrity. To enhance relevance to clinical practice and lived realities, the author team comprised TGD community members, clinicians who care for TGD youth, and guideline developers and implementers, whose combined expertise informed methodological decisions and interpretation of findings.^60^

The search protocol was developed to reflect the following *a priori* aims, with each domain assessed for all reported outcomes, including both benefits and risks, and with openness to additional categories of intervention emerging inductively from the literature:

- **Psychosocial and supportive interventions:** What is the emerging evidence on psychosocial interventions for TGD youth under 18, including different approaches to mental healthcare, peer or community support, family and parental/caregiver support or lack thereof, and school-based interventions?
- **Endocrine interventions:** What is the emerging evidence on endocrine interventions for TGD youth under 18, including puberty-pausing medication, GAHT, and other endocrine therapy?
- **Surgical interventions:** What is the emerging evidence on gender-affirming surgeries for TGD youth under 18, including masculinising chest surgery and other relevant procedures?
- **Legal, policy, and structural environments:** What is the emerging evidence on the health impacts of legal, policy, systemic and structural interventions shaping GAHC for TGD youth under 18?

### Eligibility criteria

Included publications:

- Peer-reviewed primary studies (*N* ≥ 5) reporting psychosocial or physical health outcomes of interventions for TGD youth (< 18), including interventions involving family systems, caregivers, educators, or broader public environments.
- Systematic, scoping, and narrative reviews with transparent, reproducible search and screening protocols.
- Grey-literature systematic reviews meeting equivalent methodological standards.
- Studies reporting family-level outcomes, where data specific to TGD youth were disaggregated and presented.

Excluded publications:

- Non-empirical publications, including reviews without published and reproducible protocols, commentaries, think pieces, editorials, and opinion pieces.
- Studies without psychosocial or physical health outcome data for TGD youth (< 18). Proxy-reported data from families or healthcare providers were permitted. For studies of sexual and gender minority youth, separately analysed data for at least five TGD youth were required.
- Case reports or case series with *N* < 5 were excluded. Composite or hypothetical examples were not counted towards *N*.

Given the diversity of interventions and outcomes, precise Population-Intervention-Comparator-Outcome-Time (PICOT) criteria could not be predefined. However, inclusion criteria were aligned as closely as possible to these frameworks, as shown in Table 1.^65^

**TABLE 1:** PICO(T) map of eligibility criteria for included studies.

Domain	Criteria
Population	TGD youth under 18 years old ***or*** family units including TGD youth under 18 years old. Minimum *N* = 5.
Intervention and/or exposure	Any type of psychosocial or medical care, support, policy, practice or deliberate harm delivered to or targeting TGD youth under age 18 years across all socioecological levels (individual, dyadic and/or familial, institutional, policy and/or legal, sociocultural).
Comparator and/or control	Any or none (not required).
Outcomes	Any empirically assessed health or psychosocial outcome(s).
Time	Any follow-up time frame allowed, including both prospective or retrospective assessment.
Study types	Quantitative, qualitative, mixed-methods, including prospective and retrospective designs. Systematic reviews eligible if based on transparent, reproducible search and screening protocols.

PICO(T), Population-Intervention-Comparator-Outcome-Time; TGD, transgender and gender-diverse.

All empirically assessed psychosocial and health outcomes and all empirical study designs (quantitative, qualitative, and mixed methods) were eligible. Meta-analysis was not feasible because of extreme heterogeneity in populations, interventions, and outcome measures; findings are therefore synthesised narratively. The full technical report, available at https://pathsa.org.za/resources/rapid-review-2025, adheres to Preferred Reporting Items for Systematic reviews and Meta-Analysis (PRISMA) 2020 reporting standards,^66^ with transparently reported streamlining adaptations consistent with interim published guidance for rapid reviews.^64^

### Search strategy, screening and selection

Searches were conducted on 26 November 2024, 04 December 2024, and 14 August 2025 across 12 databases via EBSCOhost (University of Pretoria), supplemented by searches of ClinicalTrials.gov and the International Standard Randomised Controlled Trial Number (ISRCTN) registry, and targeted searches for recent systematic reviews. Search date limits were set to capture records indexed between 01 January 2021 and 31 December 2025, thereby including articles indexed online ahead of their recorded publication dates. The search strategy combined [any term for trans and gender diverse identity] AND [any term for youth under 18] AND [any term for interventions related to gender identity]. Full search strings are provided in the technical report. No language restrictions were placed on the search results. Titles and abstracts of non-English sources were screened based on publisher-provided English translations whenever available and supported by Google Translate as needed.

### Data extraction and analysis

Two custom Airtable-based tools were used to extract data from primary studies and systematic reviews, capturing study characteristics, populations, interventions, outcomes, and key results. Data extraction for non-English sources was based on publisher-provided English translations in all but one case, where the full text of a Spanish-language article was translated using Google Translate. The machine-translated text was reviewed alongside the original article, with key passages verified by a Spanish-speaking research assistant. Formal *de novo* risk-of-bias or certainty grading across all individual studies was not undertaken; instead, where available, methodological appraisals and certainty assessments reported in included systematic reviews were used qualitatively to inform interpretation.

Data extraction from full-text sources was conducted by authors KL Dunkle and Ingrid Lynch. Two large language models (LLMs), ChatGPT and Perplexity, were used in a limited, supportive capacity. A structured data-extraction prompt was applied to individual PDFs to generate draft plain-text data extraction elements aligned with predefined Airtable fields. These drafts were line-checked against the original sources, corrected where necessary, and manually entered the extraction database. LLMs were instructed to flag missing, ambiguous, or uncertain data elements for targeted human review, and all errors and gaps were resolved by the human reviewers using the original reports.

LLMs were also used on an *ad hoc* basis to cross-check draft narrative descriptions of results against spreadsheets of the extracted data and source PDFs, supporting identification of potential omissions or internal inconsistencies for human correction. LLMs were not used for study screening, inclusion–exclusion decisions, formal analysis, recommendation development, conflict-of-interest statements, or contextual framing. All LLM-generated outputs were treated as provisional drafts and were reviewed, verified, and rewritten by the human author team prior to inclusion.

### Narrative synthesis of findings

Narrative syntheses drew on structured Airtable data extractions, with attention to intervention type, outcome measures, study design, sample characteristics, follow-up duration, and any certainty ratings reported in prior reviews. Findings were organised across five domains of care: psychosocial, endocrine, surgical, non-medical gender-affirming practices, and policy and legal interventions. Domain leads verified that narrative summaries accurately reflected underlying data and appropriately characterised any methodological uncertainty. The synthesis prioritised practice-relevant findings and patterns in relation to benefits, harms, and equity and access considerations, while explicitly noting where evidence was sparse, methodologically weak, or inconsistent.

Three cross-cutting interpretive lenses guided synthesis:

- Global scope and local gaps, highlighting where South African/Global South evidence remains absent.
- Equity and context, examining whether and how studies addressed intersecting axes of inequality.
- Policy and practice relevance, focusing on implications for South African clinicians, families, policymakers, and guideline developers.

Finally, to strengthen practice relevance, synthesised findings were assessed against the 2021 SAHCS GAHC Guideline.^8^ For each intervention domain, evidence was considered in terms of direction of effect, consistency, methodological quality, follow-up duration, and relevance to the South African context. Implications for existing Guideline recommendations were classified as:

- Consistent – emerging evidence aligns with current guidance.
- Refine – emerging evidence suggests that current guidance could be sharpened or made more specific.
- Challenge – emerging evidence contradicts current guidance or introduces caveats.
- New content area – emerging evidence highlights an area not addressed in current guidance.

These classifications were reviewed iteratively by the full author team, with reference to the synthesised evidence, to ensure analytic coherence and contextual appropriateness.

## Results

The final dataset comprised 200 primary studies, 29 academic systematic reviews, and four grey-literature systematic reviews, yielding 233 included sources.

**FIGURE 1:**
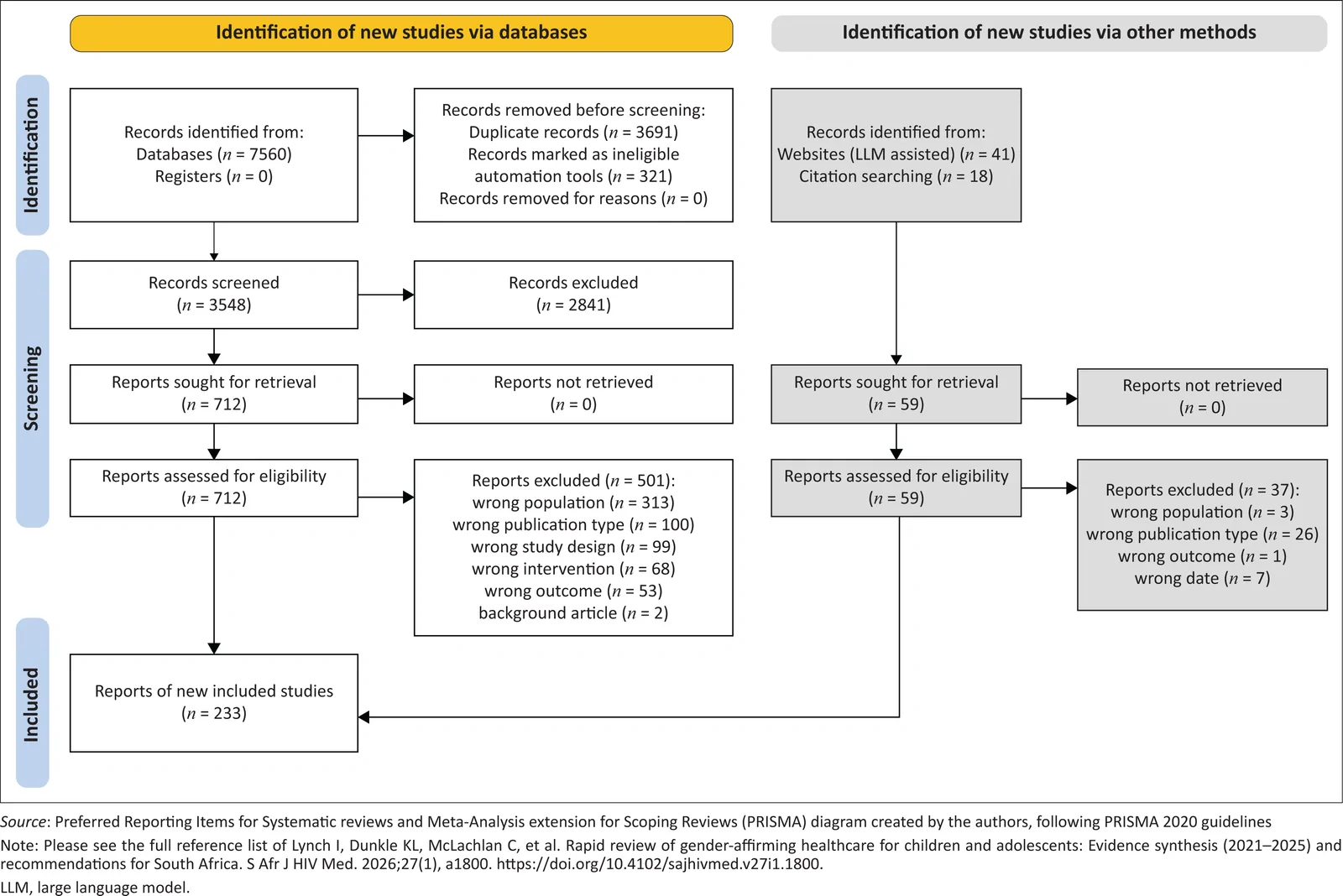
PRISMA diagram showing flow of literature searches, exclusions and inclusions of retrieved sources.

The PRISMA diagram (Figure 1) details the identification, screening, and inclusion process, including duplicate removal, automated exclusions of ineligible publication types, and abstract and full-text screening.^66,67^

The complete list of primary studies and systematic reviews, along with extracted data, is provided in the Online Supplementary Appendix (Table S-A and Table S-B).

Overall, 108 of the 200 included primary studies had been described in at least one systematic review in our dataset. We also retrieved 92 primary research articles that were *not* included in any of the prior systematic reviews identified here. The inclusion of these 92 studies increases the primary literature base considered in this report by 85.2% relative to that synthesised in the retrieved review literature. Table S-C in the Online Supplementary Appendix maps each primary study to the systematic review(s) in which it appeared and reports formal certainty appraisals, where available. For studies included in multiple reviews, certainty assessments varied according to the research questions and methods applied in each review; notes and critiques on how these were conducted appear in Table S-B and Table S-C (see Online Supplementary Appendix).

In the remainder of our analyses, prior systematic reviews were treated as evidence sources in their own right and used qualitatively to contextualise patterns and the certainty of evidence in the primary studies; however, care was taken to avoid over-emphasising primary studies appearing in multiple reviews. Synthesis and conclusions are grounded in the full source dataset, with insights from prior reviews informing interpretation rather than driving analysis.

### Psychosocial interventions and support

We identified 53 primary studies,^68,69,70,71,72,73,74,75,76,77,78,79,80,81,82,83,84,85,86,87,88,89,90,91,92,93,94,95,96,97,98,99,100,101,102,103,104,105,106,107,108,109,110,111,112,113,114,115,116,117,118,119,120^ 12 academic systematic reviews,^10,11,13,15,19,20,22,23,24,31,36,121^ and three grey-literature systematic reviews^37,38,39^ examining psychosocial aspects of care for TGD children and adolescents. The full list of primary studies is provided in Table S-D in the Online Supplementary Appendix. The evidence base is methodologically heterogeneous and dominated by observational and qualitative designs, with the majority comprising cross-sectional surveys or retrospective clinic-based analyses.^72,76,77,85,91,92,93,94,96,99,100,102,103,104,106,108,109,112,113,117,118,119,120^ A substantial proportion of studies used qualitative or mixed methods designs, drawing on interviews with young people, caregivers, families, or clinicians to explore experiences of support, identity development, barriers to access, and care processes.^70,79,80,84,87,88,90,93,98,101,105,110,111,114^

Affirming psychosocial practices such as supporting a young person’s gender expression,^19,69,83,89,91,93,94,115,117^ strengthening family and peer connectedness,^10,22,36,76,81,85,92,104,106,109^ creating safer school environments,^11,36,86,93,95,100,102,108,113,118,121^ and providing tailored psychological or digital interventions^37,68,71,73,74,75,79,120^ are associated with reductions in depression, anxiety, and suicidality, as well as improvements in resilience, sense of belonging, school participation, and daily functioning. However, access to affirming support is not evenly distributed, with nonbinary youth and racially marginalised TGD youth reporting lower levels of gender affirmation and support across social and care contexts.^91,104^ No included study or systematic review reported harms attributable to affirming psychosocial care.

By contrast, non-standard or pathologising practices – including identity-change efforts, enforced or non-clinical delays in social affirmation, or withholding affirmation – which fall outside recognised standards of psychosocial care, are consistently associated with adverse outcomes, including heightened distress, self-harm, suicidality, and deteriorating family relationships.^38,77,87,94,111^

Most studies are cross-sectional, self-reported, and concentrated in high-income settings, with relatively few longitudinal or experimental designs; these factors limit certainty of evidence in individual reports. Nonetheless, the consistency of findings across survey-based, qualitative, and mixed-methods studies, alongside diverse care contexts and outcome measures, supports psychosocial affirmation as both safe and beneficial. Taken together, findings underscore that mental health outcomes are shaped by family, school, and community contexts, and highlight the importance of supporting children and adolescents who face inconsistent or unsupportive family environments. Findings also point to the need to support youth facing overlapping social and structural stressors, and to ensure equitable access to affirming psychosocial support across diverse social, educational, and community settings.

Overall, the evidence on psychosocial interventions and support for TGD youth is consistent with the 2021 SAHCS GAHC Guideline recommendations. Table 2 summarises key implications for guideline development, mapping synthesised evidence against existing guidance and highlighting areas of support and refinement.

**TABLE 2:** Summary of evidence and guideline implications for psychosocial care (2021–2025).

Domain	Recommendation (SAHCS 2021)	Implication (2021–2025 evidence)
1. Informed consent	For children under 12, consent from parents/guardians with child assent; for adolescents ≥ 12, participatory consent involving adolescent and caregiver(s); disclose risks/benefits.	**Consistent** → Evidence supports consent as a youth-centred, affirming process. **Refine** → Emphasise neurodiversity- informed adaptations (clear structure, accessible communication).
2. Social transition	Affirmed names, pronouns, clothing, and gender expression should be supported when requested; processes should be discussed with families/schools.	**Consistent** → Evidence shows strong protective effects of affirmation, stability of early transitions, and harms when support is delayed. **Refine** → Emphasise that both childhood and adolescent transitions are safe when affirmed, and that additional support might be required in hostile school or community environments.
3. Family involvement	Family participation improves outcomes; interventions may include psychoeducation, counselling, and resource-linking for caregivers and extended family.	**Consistent** → Evidence supports family affirmation as the key protective factor. **Refine** → Emphasise psychoeducation and supportive interventions that help families move through fears/uncertainties, and the importance of proactive safety-planning if family rejection occurs.
4. School and community engagement	MHPs should support TGD youth in navigating schools and communities, advocate for safe and inclusive environments, and provide psychoeducation to stakeholders.	**Consistent** → Evidence reinforces the protective role of inclusive school climates, safe bathrooms, supportive teachers, and access to affirming counselling and confidential school-based spaces. Harms are linked to victimisation and exclusion rather than affirmation.
5. Mental health assessment and support	MHPs should assess/address co-occurring conditions (depression, anxiety, suicidality, ASD) without pathologising identity.	**Consistent** → High rates of distress are linked to victimisation/rejection, not identity. Affirming interventions show benefit. **Refine** → Explicitly recommend autism-informed practices (structured sessions, sensory adaptations) and recognise distress arising from care delays or prolonged waitlists.
6. Affirmation and non-pathologisation	Care must be affirming, non-pathologising, and avoid gatekeeping; centre the adolescent’s lived experience.	**Consistent** → Affirmation reduces suicidality and distress, while restrictive or ‘watchful waiting’ approaches that withhold affirmation cause harm. **Refine** → Emphasise that non-clinical enforced delays undermine well-being and are inconsistent with affirming care.
7. Enabling affirming environments	MHPs should advocate for TGD youth’s rights, contribute to policy/institutional reforms, and promote safe environments.	**Consistent** → Supportive family, school, and community environments are strongly protective. **Refine** → Emphasise anticipating minority stress in hostile social environments and the need to address inequities faced by multiply marginalised youth.
8. Contraindicated approaches	Practices such as gender identity and expression change efforts (‘conversion therapy’), pathologisation, or non-clinical delays in care are harmful and contraindicated.	**Consistent** → Evidence confirms these practices increase distress, self-harm and suicidality. **Refine** → Explicitly name contraindicated practices.
9. Equity and access	Not explicitly addressed in the 2021 guideline.	**New** → Evidence highlights persistent inequities in affirmation and safety for youth facing intersecting forms of exclusion – including nonbinary young people and those in unsupportive families or schools. Future guidance should explicitly incorporate equity considerations.

MHP, Mental Healthcare Practitioner; TGD, transgender and gender-diverse; ASD, Autism spectrum disorder; SAHCS, South African HIV Clinicians Society.

### Endocrine interventions

We identified 23 primary studies,^122,123,124,125,126,127,128,129,130,131,132,133,134,135,136,137,138,139,140,141,142,143,144^ three academic reviews,^26,28,33^ and one grey-literature report^39^ with focused evaluation of puberty-pausing medication; 28 primary studies,^145,146,147,148,149,150,151,152,153,154,155,156,157,158,159,160,161,162,163,164,165,166,167,168,169,170,171,172^ and two academic reviews^25,32^ with focused evaluation of GAHT; and 53 primary studies,^78,87,88,153,154,155,156,173,174,175,176,177,178,179,180,181,182,183,184,185,186,187,188,189,190,191,192,193,194,195,196,197,198,199,200,201,202,203,204,205,206,207,208,209,210,211,212,213,214,215,216,217,218,219,220^ 11 academic reviews^9,11,12,16,17,18,21,27,29,34,35^ and three grey-literature reports^37,38,40^ with joint or overlapping evaluation of puberty pausers and GAHT (see Online Supplementary Appendix: Table S-E, Table S-F and Table S-G).

The literature shows a consistent pattern: endocrine interventions for TGD adolescents generally achieve their intended physiological effects^25,32,33,37,38,40,124,126,203^; adverse events are typically anticipated, predominantly mild, and manageable under routine monitoring in specialist care, with specific adverse events described in relation to individual interventions below.^123,124,131,146,150,173^ Mental-health outcomes are broadly stable or improved.^122,127,142,166,209^ Although the evidence base is methodologically constrained and concentrated in high-income clinical settings, available data provide a short- to medium-term picture of effectiveness and safety.^9,21,27,34,35^

Puberty-pausing medication reliably halts unwanted pubertal development,^39,124,126,130,136,142,143^ and GAHT induces expected masculinising or feminising changes.^25,32,37,38,150^ Endocrine and metabolic parameters typically remain within clinically acceptable ranges under supervised care.^124,140,150,210^ Bone-density reductions during pubertal suppression are common and well-described,^125,132,137,195^ and typically show partial or substantial recovery following subsequent GAHT or endogenous puberty, especially when progression to hormones is timely and aligned with clinical indicators.^125,175,197,203,207,213^ Serious adverse events are rare across endocrine pathways, with no consistent pattern of irreversible harm.^149,173,204,216,220^

Mental-health outcomes are heterogeneous at the individual level, yet broadly stable-to-favourable across cohorts. Adolescents who access puberty suppression and/or GAHT generally show reductions in depressive symptoms and suicidality, alongside improved appearance congruence and functioning, compared with their own baseline and with peers who want but cannot access treatment.^160,161,164,166,181,194,209^ Worsening mental-health trajectories are more consistently associated with delayed or restricted access to care than with endocrine treatment,^166,209^ with GAHT linked to lower risks of suicidality-related emergency or inpatient care and to fewer mental-health diagnoses and psychiatric hospitalisations following treatment initiation.^192,200^

Longitudinal data indicate high continuation into adulthood and very low reported regret among adolescents who commence endocrine care.^153,154,156,212,213^ Where discontinuation occurs, it is very often linked to external or structural factors (such as access barriers, service disruptions, costs, insurance denials, family stressors, or bullying), completion of desired physical changes, or evolving identity-related needs, rather than to treatment-related side effects or regret.^16,153,154,163,184^

Although menstrual suppression as gender-affirming care is not explicitly addressed in the 2021 SAHCS GAHC Guideline, review of evidence from five primary sources^221,222,223,224,225^ (Online Supplementary Appendix: Table S-H) suggests that it is a safe, effective, and highly valued component of care for TGD adolescents who menstruate. Reported outcomes include high rates of amenorrhoea, reduced pain and menstrual-related dysphoria, and high overall satisfaction, with mild and infrequent adverse events.^221,222,223,224,225^

Fertility preservation was addressed in 16 primary sources^226,227,228,229,230,231,232,233,234,235,236,237,238,239,240,241^ (Online Supplementary Appendix: Table S-I), one academic systematic review,^30^ and two grey-literature reviews.^37,38^ Although gamete preservation appears feasible and generally safe for adolescents, interest is typically low, and accessibility for those who are interested is substantially constrained by cost, procedural dysphoria, and timing of referral.^37,38,226,227,229,230,231,232,233,234,235,236,237,238,239,240,241^ These factors highlight the importance of early, developmentally appropriate, and iterative fertility counselling.

Overall, the evidence aligns with the 2021 SAHCS GAHC Guideline recommendations. Table 3 and Table 4 summarise key implications for guideline development for puberty pausing and GAHT, respectively, mapping synthesised evidence against existing recommendations. Table 5 outlines implications for future guideline development related to menstrual suppression.

**TABLE 3:** Summary of evidence and guideline implications for puberty-pausing medication (2021–2025).

Domain	Recommendation (SAHCS 2021)	Implication (2021–2025 evidence)
1. Indications and eligibility	Offer GnRHa from Tanner stage 2 for adolescents with persistent gender incongruence to relieve dysphoria, prevent unwanted pubertal changes, and support well-being.	**Consistent** → Evidence confirms reliable suppression and short-term safety. **Refine** → Earlier initiation is associated with more favourable physical and psychosocial trajectories; non-clinical delays reduce effectiveness and increase distress.
2. Initiation and consent	Initiate after MDT assessment confirming gender incongruence and capacity for informed consent; adolescents ≥ 12 may consent independently; caregiver involvement encouraged.	**Consistent** → Current MDT-based framework remains appropriate; combined-pathway evidence shows that coordinated endocrine–mental-healthcare supports clearer counselling and smoother decisions about timing and possible GAHT sequencing.
3. Family / caregiver involvement	Collaborative approach improves outcomes; lack of family support should not preclude access.	**Consistent** → Evidence shows that family support enhances well-being. Combined-pathway data suggest better mental-health trajectories when adolescents have family engagement during both PS and GAHT stages, underscoring the importance of family counselling where feasible.
4. Clinical oversight	Care should be managed by paediatric endocrinologist or trained provider using available GnRHa agents.	**Consistent** → Evidence reinforces the need for skilled oversight.
5. Information and counselling	Provide clear information on benefits, risks, and reversibility.	**Consistent** → Evidence supports the importance of clear, anticipatory counselling about expected effects, reversibility, and expected timing of potential subsequent GAHT.
6. Fertility	Counsel before initiation; early suppression can preclude gamete preservation.	**Consistent** → Evidence remains limited but coherent, and supports early, developmentally tailored counselling. Semen and oocyte cryopreservation are feasible and generally safe, but uptake is low due to procedural dysphoria, high cost, and late referral.
7. Monitoring – Psychosocial well-being	Follow-up should include psychosocial monitoring.	**Consistent** → Evidence indicates stable or improved mental health during PS, with no signals of harm. Combined-pathway findings show additional psychological benefit when progression to GAHT is timely and uninterrupted.
8. Monitoring – Bone health and growth	Monitor bone health and growth during follow-up.	**Consistent**→ Evidence shows transient BMD reductions (especially lumbar spine) and slowed growth during PS, with recovery after GAHT. **Refine** → Earlier pubertal stage at initiation is linked to more favourable growth and bone-density trajectories, while later initiation and prolonged GnRHa monotherapy may constrain recovery, underscoring the need for DXA monitoring where clinically indicated.
9. Monitoring – Body composition and metabolic indicators	Not explicit in 2021 guideline.	**Refine** → PS increases fat mass and reduces lean mass, which generally normalises after GAHT. Include anticipatory guidance on these expected changes, alongside counselling on physical activity and nutrition to support bone health and cardiometabolic well-being.
10. ASD considerations	Use concrete communication; involve ASD-experienced clinicians.	**Consistent** → No new PS-specific evidence. Maintain guidance.
11. Equity and access	Not explicitly addressed in 2021 guideline.	**New** → Structural barriers (delays, insurance, geographic inequities) affect timing and outcomes. Combined-pathway evidence shows delays between PS and GAHT can worsen mental-health trajectories. Add explicit guidance on equitable access, provincial provision, and appeal pathways.

SAHCS, South African HIV Clinicians Society; GnRHa, Gonadotropin-Releasing Hormone Agonist; MDT, Multi-disciplinary team; GAHT, gender-affirming hormone therapy; PS, Puberty supression; BMD, Bone mineral density; DXA, Dual energy X-ray absorptiometry; ASD, Autism spectrum disorder.

**TABLE 4:** Summary of evidence and guideline implications for gender-affirming hormone therapy (2021–2025).

Domain	Recommendation (SAHCS 2021)	Implication (2021–2025 evidence)
1. Indications and eligibility	Initiate GAHT for adolescents with persistent incongruence, desire for hormonal change, and capacity for informed consent.	**Consistent** → Evidence shows GAHT reliably induces intended changes and improves well-being. Combined-pathway evidence shows continuity of benefit when GAHT follows timely PS.
2. Consent	From age 12, competent adolescents may consent independently; partially irreversible effects require careful counselling.	**Consistent** → Maintain detailed counselling. Combined-pathway data reinforces the need to discuss sequencing, timing, and irreversible features.
3. Decision-making	Clinicians and MHPs should confer on readiness; parental input improves outcomes.	**Consistent** → MDT approach remains appropriate. Combined-pathway evidence highlights the value of coordinated endocrine-mental healthcare during transition from PS to GAHT.
4. Timing	Timing should be individualised, considering prior PS, growth potential, family support, and risks of delay.	**Refine** → Structural or administrative delays are associated with worsening mental-health symptoms. Combined-pathway studies indicate improved well-being and social functioning when progression from PS to GAHT is uninterrupted. Service planning should define and support timely access.
5. Regimens (including non-binary care)	Gradual dose escalation in younger adolescents; tailor to goals, including for non-binary youth.	**Consistent** → Goal-based titration remains appropriate. Combined-pathway data show that earlier PS may shape later outcomes (height, hip geometry). Continue supporting flexible dosing for non-binary clients.
6. Fertility	Provide counselling before GAHT on irreversible effects and preservation options.	**Consistent** → Evidence supports early, realistic fertility counselling. Gamete preservation is often feasible when GAHT is paused for several months, but adolescent data remain limited and uptake is low, partly due to cost, procedural dysphoria, and late referral.
7. Contraception	GAHT is not contraceptive; discuss contraception where pregnancy is possible.	**Consistent** → Maintain guidance.
8. Concurrent health needs	Manage co-existing conditions alongside GAHT; do not delay access unnecessarily.	**Consistent** → Evidence supports this approach; there is no evidence that supports delaying GAHT for common concurrent conditions.
9. Monitoring – Bone health and growth	Monitor bone health and growth.	**Refine** → Combined-pathway evidence shows that BMD typically declines during GnRHa and increases after GAHT, with generally favourable long-term recovery, including stronger catch-up on testosterone. Transfeminine adolescents may have persistent lumbar-spine deficits, underscoring the need for ongoing DXA monitoring, adequate oestrogen dosing, vitamin D assessment, and avoidance of prolonged suppression without progression to either GAHT or endogenous puberty, as indicated.
10. Monitoring – Body composition and metabolic indicators	Monitor lipids, glucose, liver enzymes, and other metabolic markers.	**Consistent** → Predictable metabolic shifts occur that are consistent with the affirmed gender and are rarely clinically significant. Combined-pathway data show no additional metabolic safety concerns when transitioning from PS to GAHT.
11. Monitoring – Cardiovascular/thrombosis	Include cardiovascular risk assessment.	**Consistent** → Adolescents on GAHT do not show new cardiovascular safety signals; thrombotic events are rare. Combined-pathway studies report small QTc changes and transient blood-pressure shifts that remain within normal ranges, including among youth on psychotropic medications.
12. Monitoring – Psychosocial outcomes	Monitor psychosocial well-being and satisfaction.	**Consistent** → Evidence demonstrates improved well-being and high satisfaction during GAHT. Combined-pathway studies show very high continuation, rare regret, and stable or improved mental-health trajectories from PS → GAHT.
13. Equity and access	Not explicit in 2021 guideline.	**New** → Structural inequities (funding, geography, age-based restrictions, service delays) shape treatment timing and outcomes. Combined-pathway findings show that delays between PS and GAHT worsen psychosocial symptoms. Add explicit equity guidance: provincial scaling, public-sector funding, telehealth access, and accountability mechanisms for wait times.

GAHT, gender-affirming hormone therapy; PS, Puberty supression; MHP, Mental Healthcare Provider; MDT, Multi-disciplinary team; GnRHa, Gonadotropin-Releasing Hormone Agonist; DXA, Dual energy X-ray absorptiometry; QTc, corrected QT interval on an electrocardiogram.

**TABLE 5:** Summary of evidence and guideline implications for menstrual suppression (2021–2025).

Domain	Recommendation (SAHCS 2021)	Implication (2021–2025 evidence)
1. Indication and purpose	Mentioned only as an option when bleeding persists on testosterone or when clients do not wish to use testosterone.	**New** → Evidence shows menstrual suppression functions as a gender-affirming intervention that reduces menstrual-related dysphoria and pain. Recognise suppression as a standalone component of gender-affirming care, not only an adjunct to testosterone.
2. Eligible methods	Leuprolide, medroxyprogesterone acetate, anastrazole may be considered.	**Refine** → High amenorrhoea rates and favourable safety profiles demonstrated across progestin-based methods and LARC (LNG-IUD, etonogestrel implant). Expand recommended evidence-based method options and include expected effectiveness.
3. Counselling and informed consent	No adolescent-specific guidance; general advice only.	**New** → Provide anticipatory, developmentally appropriate counselling on expected bleeding patterns, onset of amenorrhoea, side-effect profiles, and integration with future GAHT or reproductive-health planning. Clarify that menstrual suppression does not impair long-term fertility and may support adolescents who wish to preserve future reproductive options. Address potential dysphoria linked to procedures (e.g. pelvic exams, IUD insertion).
4. Psychosocial outcomes	Not addressed.	**New** → Evidence shows reductions in menstrual-related dysphoria, improved daily functioning, and high satisfaction once amenorrhoea is achieved. Highlight psychosocial benefits in the guideline.
5. Safety and adverse events	Not addressed.	**New** → Evidence shows generally mild, manageable adverse effects and no serious complications. Reinforce routine monitoring and anticipatory side-effect counselling.
6. Equity and access	Not addressed.	**New** → Address access barriers (cost, method availability, procedural discomfort, and variable provider experience with gender-affirming menstrual suppression) to support equitable provision across public-sector services.

SAHCS, South African HIV Clinicians Society; LARC, Long-Acting Reversible Contraception; LNG-IUD, Levonorgestrel Intrauterine Device; GAHT, gender-affirming hormone therapy.

### Surgical interventions

We identified 10 primary studies reporting outcomes of gender-affirming surgery in adolescents,^242,243,244,245,246,247,248,249,250,251^ and 15 primary studies in which surgery was examined within broader gender-affirming care trajectories that also included endocrine and/or psychosocial care^163,184,196,213,228,252,253,254,255,256,257,258,259,260,261^ (Online Supplementary Appendix: Table S-J1 and Table S-J2). Two grey-literature systematic reviews assessed surgical outcomes in youth.^37,38^

Evidence for patients under age 18 is limited in volume but highly consistent, focusing almost entirely on masculinising chest reconstruction, which is the only gender-affirming surgery routinely accessed by adolescents.^242,243,244,245,246,247,248,249,250,251^ A very small number of other procedures (e.g. hysterectomy, vaginectomy, facial feminisation) are reported in United States national registry data, but are vanishingly rare in under-18s.^245^ Within multidisciplinary programmes, masculinising chest surgery shows very low complication and revision rates, with safety profiles favourably comparable to those observed in adults or cisgender youth undergoing analogous chest procedures.^242,244,245,249,250^

Psychosocial outcomes of gender-affirming surgery are consistently positive in the short to medium term, including improved body image, reduced dysphoria, and increased participation in social, educational, and physical activities, alongside high levels of patient satisfaction.^37,38,243,247,250,251^ Reported regret is rare, including in the few longer-term follow-up studies that are available.^37,247,249,250^ Studies examining surgical interventions within broader gender-affirming care trajectories report similarly favourable safety and psychosocial outcomes, reinforcing findings from surgery-specific cohorts.^163,184,196,213,228,252,253,254,255,256,257,258,259,260,261^ Adolescents considering or recovering from surgery benefit from ongoing care from multidisciplinary teams, affirming family support, and peer networks.^228,256,259^ These findings reinforce the importance of situating surgical interventions within broader trajectories of gender affirmation, including ongoing psychosocial support.

Findings are consistent with the 2021 GAHC Guideline recommendations. Table 6 summarises key implications for guideline development and identifies areas for potential refinement.

**TABLE 6:** Summary of evidence and guideline implications for adolescent gender-affirming surgery (2021–2025).

Domain	Recommendation (SAHCS 2021)	Implication (2021–2025 evidence)
1. Eligibility and decision-making	Surgical eligibility and decision-making should be determined by a multidisciplinary team, including mental-health professional(s), surgeon(s), the adolescent, and parents/legal guardians.	**Consistent** → All adolescent surgeries in the identified evidence occurred within specialist multidisciplinary services and were embedded in broader GAHC pathways. Outcomes were favourable in these settings. No evidence supports modifying multidisciplinary eligibility processes; findings reinforce the importance of team-based, longitudinal, individualised care.
2. Indications and type of surgery in adolescence	For adolescents, GAS is most commonly limited to masculinising chest reconstruction for those mature enough to require and benefit from surgery; other procedures are usually deferred to adulthood.	**Consistent** → Evidence in minors is almost entirely limited to masculinising chest surgery, with high satisfaction and very low regret in small, mostly single-centre cohorts with low- to very-low-certainty evidence, though point estimates for regret are consistently close to zero. Insufficient data exist to expand adolescent indications, but findings support maintaining access where clinically indicated.
3. Preoperative requirements	Preoperative requirements include a thorough informed-consent process and at least one referral letter from a mental-health provider familiar with gender-affirming healthcare.	**Refine** → Low complication rates, high satisfaction, and very low regret occur in settings where careful assessment and preparation are standard. At the same time, evidence of unmet need and distress from cost, waitlists, and policy restrictions suggests preventing and avoiding non-medical delays beyond those required for safe, well-informed decision-making.
4. Consent	Consent requires the adolescent to be over 12, sufficiently mature, cognitively capable, and legally assisted by a parent/legal guardian. Irreversibility must be emphasised.	**Consistent** → No included studies examined consent processes, and South African legal standards remain unchanged. Current guidance is appropriate, especially as surgery is typically only offered to older adolescents. Emerging evidence affirms the value of clear communication and expectation-setting.
5. Autonomy and well-being	The autonomy and well-being of the adolescent must be respected and central to decisions.	**Refine** → Improvements in dysphoria, body image, daily functioning, and social participation, alongside very low regret, underscore the importance of centring adolescent goals. Evidence that non-clinical delays contribute to distress reinforces balancing caution with respect for adolescents’ bodily autonomy through shared decision making that actively incorporates adolescents’ stated priorities.
6. Psychosocial support	Comprehensive psychosocial support before and after surgery is strongly recommended; ongoing support is especially important for adolescents with neurodevelopmental or psychological challenges.	**Refine** → Surgery alleviates major sources of distress but does not address broader minority stress or structural barriers. Evidence emphasises structured, ongoing mental-health, family, peer, and school-based support to sustain gains in well-being while addressing ongoing minority stressors.
7. Post-surgical care, safety and complications	Post-surgical care should include psychological support, physiotherapy, and peer/community support resources.	**Refine** → Available cohort and systematic-review evidence from predominantly single-centre, observational studies demonstrates low complication rates in adolescents, with serious events rare and rates comparable to, or lower than, adults or cisgender adolescents undergoing analogous procedures. Guidelines could more explicitly acknowledge this favourable safety profile while reinforcing the need for structured follow-up and ongoing psychosocial care.
8. Equity and access	Access is constrained by limited resources, long public waitlists, and high private-sector costs.	**Refine** → Evidence from cohort and qualitative studies shows structural barriers (cost, geography, waitlists and policy restrictions) drive treatment delays, unmet need and psychological distress. Guidelines could incorporate explicit equity-focused recommendations: reducing non-clinical delays, scaling public-sector capacity, transparent waitlist management, and advocacy for funding and rights-based accountability.

SAHCS, South African HIV Clinicians Society; GAHC, gender-affirming healthcare; GAS, Gender-affirming surgery.

### Non-medical gender-affirming practices

We identified one large online survey^262^ (Online Supplementary Appendix: Table S-K) and one narrative review^14^ addressing non-medical gender-affirming practices, including binding, tucking, packing, and padding. Although the evidence base is limited, findings indicate that these practices are meaningful tools through which TGD adolescents manage gender dysphoria, navigate public and social spaces, and feel more at ease in their bodies.^14,262^ For many adolescents, these practices are partly driven by limited or delayed access to endocrine and/or surgical GAHC. The available evidence does not indicate serious health risks associated with packing or padding. Physical discomfort related to binding and tucking appears to be common but manageable when TGD youth have access to safer materials and practical guidance.^14,262^

### Policy and legal interventions

Although policy and legal interventions are not addressed as a standalone domain in the SAHCS GAHC Guideline (2021), this rapid review identified eight studies examining the health impact of laws, regulations, and/or administrative systems on TGD youth^111,261,263,264,265,266,267,268^ (Online Supplementary Appendix: Table S-L). No systematic reviews were identified.

Across both qualitative and quantitative studies, restrictive laws and systemic barriers – including outright healthcare bans, regulatory constraints, and administrative obstacles – are consistently associated with poorer mental-health outcomes, including heightened depression, anxiety, suicidality, and social isolation among TGD youth.^111,261,263,264,265,266,267,268^ These associations are observed following both enacted policies and periods of policy uncertainty, suggesting that anticipatory stress and disruption to care pathways contribute to harm.

Conversely, affirming and protective policy environments, including anti-discrimination protections, inclusive institutional guidance, and accessible legal gender recognition, are associated with improved mental-health indicators and, in some analyses, lower substance use and other risk behaviours, as well as enhanced overall well-being.^261,265,267^

Parental and caregiver accounts mirror these findings, describing how proposed or enacted care bans intensified depression, anxiety, suicidality, and family distress, particularly where policies disrupted ongoing treatment or created prolonged uncertainty.^263,264,266^ Across studies, families reported increased emotional burden, logistical strain, and fear related to maintaining access to care within hostile or unstable policy environments.^263,264,266^

## Discussion

The global evidence base on GAHC for TGD children and adolescents has expanded rapidly since the publication of the SAHCS GAHC Guideline in 2021. Across psychosocial, endocrine, surgical, non-medical and policy interventions, the evidence from 2021 to 2025 demonstrates a consistent pattern across diverse study designs and contexts: when TGD children and adolescents receive GAHC within supportive familial, social, educational, clinical, and policy environments, outcomes are generally stable or improved, and harms are rare. Conversely, the evidence indicates that non-clinical delays in providing GAHC, as well as hostile familial, social, educational and policy environments, are associated with worsening distress and poorer mental-health outcomes. Across intervention domains, studies conducted within multidisciplinary care settings consistently report favourable safety and psychosocial outcomes, highlighting the importance of coordinated and integrated care across service levels.

Across intervention domains, the emerging evidence aligns with the 2021 SAHCS GAHC Guideline, reinforcing its core recommendations and ethical foundations, while also pointing to areas where future guideline refinements are warranted.

### Strengths and limitations of this review

The review has several important strengths. It was conceived and led from South Africa by a gender and sexually diverse team with combined methodological, clinical, and lived-experience expertise. This positionality informed decisions about contextualisation and equity, ensuring that the synthesis remains grounded in the legal, social, and service realities of TGD youth in South Africa rather than importing assumptions from high-income settings. To our knowledge, this is the first comprehensive synthesis of GAHC evidence for people under 18 from the Global South.

The search strategy was broad and intentionally inclusive. We conducted a comprehensive multi-database search via EBSCOhost with no language restrictions, deliberately incorporating both current and pathologising terminology for TGD identities and interventions. Cross-mapping showed that 92 of 200 primary studies in our dataset had not appeared in any of the systematic reviews identified, substantially extending the evidence base available for synthesis and highlighting limitations in existing reviews. Data extraction and synthesis followed a structured and transparent process.

As is typical for rapid reviews, several methodological streamlining decisions were made. The review was not prospectively registered. After an initial dual-screening calibration phase, title and abstract screening proceeded with single review, with borderline or uncertain records flagged for second-reviewer input at abstract or full-text stage. Full-text screening and data extraction were not conducted in duplicate. We did not conduct a *de novo*, outcome-by-outcome risk-of-bias or GRADE/CERQual certainty assessment; instead, we drew on existing appraisals from previous systematic reviews where available and treated them as a secondary interpretive resource rather than as formal ratings for this review. No meta-analysis was attempted because of the extreme heterogeneity of designs, interventions, and outcomes. These choices are consistent with published guidance on rapid reviews, but they inevitably reduce the granularity of quality assessment and may increase susceptibility to subjective judgement compared with a fully resourced systematic review.^60,63,64,269,270^

Nonetheless, the breadth of the search, the systematic cross-linking of primary studies and prior reviews, the large number of newly identified studies, and the structured narrative synthesis conducted by a multidisciplinary author team support the robustness and practical value of the findings. The review should therefore be understood as a transparent, contextually grounded rapid synthesis rather than a definitive systematic review, and its conclusions should be interpreted with reasoned discernment and attention to the underlying evidentiary limitations.

### Strengths and limitations of the evidence base covered in this review

The evidence assembled in this rapid review – spanning 200 primary studies, 29 academic systematic reviews, and four grey-literature reports – demonstrates a consistent pattern across psychosocial, endocrine, surgical, non-medical, and policy interventions. When TGD young people access affirming psychosocial support, timely endocrine care, and, for older adolescents who require it, surgical interventions, improvements are seen in mental health, dysphoria, and day-to-day functioning. Serious adverse events are rare. Systematic reviews published since 2021, including those commissioned in politically conservative policy environments, echo these findings and report no evidence of population-level harm.^9,10,11,12,13,14,15,16,17,18,19,20,21,22,23,24,25,26,27,28,29,30,31,32,33,34,37,38,39,40,121^ This consistency across diverse study designs and outcome domains strengthens confidence in the overall direction of effect, even where formal certainty ratings remain low.

Important limitations in the evidence remain. Most primary studies are observational and/or clinic-based, with small, non-representative samples, short follow-up, and limited geographic diversity. Randomised or quasi-experimental designs are scarce; they are rarely feasible or ethical in this area of care.^271^ Evidence for endocrine and surgical care currently relies heavily on retrospective chart reviews, with variable data quality, inconsistent outcome recording, and limited use of patient-reported measures. Frequent access to multiple and concurrent gender-affirming intervention types by individual TGD youth constrains precision in assessing the impact of particular interventions and requires methodological advancement in the field to disentangle causality while preserving patient autonomy.

Systematic reviews reflect these constraints. Across reviews, varying assessment approaches consistently rate the certainty of evidence as low to very low, because of design limitations, small sample sizes, overlapping patient populations and interventions, short follow-up periods, and narrow geographic scope, rather than conflicting findings. These gaps underscore the need for more robust, longer-term, and inclusive research from the Global South, and South Africa in particular, to better characterise evolving health needs and support equitable, contextually responsive health-system planning.

### Clinical and policy decision-making in the context of low-certainty evidence

The certainty of the evidence surrounding GAHC for youth must be understood within the broader reality that low- and very low-certainty evidence underpins a substantial proportion of medical practice overall.^272,273,274^ This is particularly true in paediatrics, where ethical and logistical constraints often preclude randomised trials and contribute to persistent inconsistencies across the evidence base, even for common childhood conditions such as asthma and epilepsy.^274,275^

The recently updated GRADE Evidence-to-Decision (EtD) framework directly acknowledges these realities. As clarified in the 2025 Core GRADE 7 update,^276^ low-certainty evidence typically warrants a conditional recommendation, which is an explicit signal that clinicians must engage meaningfully in shared decision-making that centres patient values and preferences. Gordon Guyatt, leading architect of the GRADE framework and co-author of systematic reviews included in this synthesis,^25,26^ has explicitly cautioned against the misuse of GRADE in policy advocacy against GAHC for youth.^277^ Guyatt and colleagues^277^ emphasise that low-certainty evidence should not be treated as justification for withholding care, but rather as a prompt for careful clinical decision-making that centres patient values, noting:

It is profoundly misguided to cast health care based on low-certainty evidence as bad care or as care driven by ideology, and low-certainty evidence as bad science. Many of the interventions we offer are based on low-certainty evidence […]. Thus, forbidding delivery of gender-affirming care and limiting medical management options on the basis of low-certainty evidence is a clear violation of the principles of evidence-based shared decision-making and is unconscionable.^277^

In South Africa, this is not simply a methodological consideration but a constitutional and statutory imperative. Section 129 of the *Children’s Act*^278^ requires respect for adolescents’ evolving capacity to participate meaningfully in decisions about their own healthcare. Where the overall evidence base offers low scientific certainty yet demonstrates consistent patterns across multiple domains and outcomes, the GRADE EtD framework requires clinicians to centre patients’ values, preferences, and lived experience – not to impose blanket restrictions or personal disapproval framed as clinical caution. This approach aligns with the informed-consent model endorsed in South African and international clinical guidelines on GAHC for youth.^1,8^

### Implications and recommendations for South Africa

In addition to the domain-specific comparisons with the 2021 SAHCS GAHC Guideline, the evidence synthesised in this review has broader clinical and policy implications that cut across intervention areas and highlight key principles for delivering safe, effective, and contextually grounded care:

- Affirmation is central to safe and effective care, reflecting both clinical evidence and South Africa’s constitutional and rights-based obligations.
- Multidisciplinary, coordinated care models that integrate medical, psychosocial, and community-based components, are associated with better outcomes across intervention domains, supporting continuity and safety of care over time.
- Structured and ongoing support for families and caregivers is essential, as affirming family environments are consistently linked with improved mental-health outcomes, sustained engagement in care, and enhanced daily functioning.
- Affirming psychosocial support should be integrated across all aspects of care, including clinical encounters, family engagement, and school or community settings, rather than treated as an ancillary component.
- Restrictive policies and administrative environments, including opaque referral structures, regulatory barriers, and medical-aid exclusions that limit affordability, are associated with delayed or disrupted access to care and related harms.
- Supportive and protective policy environments – such as those that prohibit discrimination, provide clear clinical guidance, and support continuity of care – are associated with improved population-level well-being, including lower distress and suicidality, greater resilience, and reduced reliance on acute mental-health and emergency services.
- Health system constraints, including medication stock-outs, long waiting lists, and uneven geographic distribution of clinical expertise, require targeted policy and planning responses, as these barriers leave many adolescents without feasible routes into care.
- Equitable financing mechanisms are needed, as financial exclusion disproportionately limits access to care for economically marginalised families and entrenches inequities across provinces, and between public and private healthcare sectors.
- Strengthening adolescent-friendly services and integrated care pathways enables many non-specialised components of gender-affirming care – including psychosocial support for young people and their families, menstrual suppression, trauma-informed counselling, management of co-occurring mental-health conditions, and safe referral pathways – to be delivered within routine adolescent health and mental-health services. This approach aligns with national priorities in HIV/SRHR, youth mental health, and adolescent-friendly service delivery.
- Timely access to GAHC is a matter of health equity and cost-effective prevention, given the documented harms associated with delayed care and its downstream impacts on schooling, psychological distress, and emergency mental-health utilisation.

Priorities for future research include expanding the evidence base in low- and middle-income countries, strengthening rigour and long-term follow-up, improving the visibility of non-binary adolescents in research, investigating intersecting structural determinants of health, extending evidence on menstrual suppression and adolescent fertility pathways, and deepening TGD youths’ involvement in co-produced and participatory research.

## Conclusion

This rapid review provides empirical support that GAHC for TGD youth is evidence-informed, associated with improved well-being, prevents harm, and promotes healthier developmental and mental-health trajectories when delivered within supportive social, familial, clinical, and policy environments. The review affirms the evidentiary foundations of the existing South African GAHC Guideline and identifies opportunities to strengthen its implementation within South Africa’s legal, historical, and health-system context. While most available studies are observational, this reflects the ethical and methodological realities of paediatric research on interventions that cannot be logistically or ethically randomised or withheld. The consistency of findings across diverse settings therefore provides compelling, real-world evidence of effectiveness and safety.

Ultimately, this work reflects South Africa’s constitutional values, including dignity, equality, and the right to access healthcare without discrimination. South Africa has a long-standing tradition of protecting marginalised communities, guided by principles of collective care, justice and equity. Ensuring timely affirming care for TGD youth is both clinically sound and constitutionally grounded, representing a continuation of this legacy and a commitment to safeguarding the well-being of every young person entrusted to collective care.
